# PEG/Folic Acid Coloaded Er:Y_2_O_3_ Upconversion Nanoparticles: Enhanced Internalization and Potential for High-Contrast Bioimaging in HCT-116 Cells

**DOI:** 10.1021/acsomega.6c00386

**Published:** 2026-03-04

**Authors:** Ester Butera, Regina Maria Chiechio, Angela Caponnetto, Carmen Ferrara, Cinzia Di Pietro, Paolo Musumeci, Riccardo Reitano, Luca Lanzanò, Francesco Ruffino, Salvatore Petralia, Giovanni Arena, Carlotta Cosentino, Valerie Marchi, Annalinda Contino, Giuseppe Maccarrone

**Affiliations:** † Dipartimento di Scienze del Farmaco, 9298 Università di Catania, Via Santa Sofia 64, Catania 95125 Italy; ‡ Dipartimento di Fisica e Astronomia “Ettore Majorana”, 9298 Università di Catania, Via Santa Sofia 64, Catania 95123, Italy; § Dipartimento di Scienze Biomediche e Biotecnologiche, Sezione di Biologia e Genetica “G. Sichel”, 9298 Università di Catania, Via S. Sofia, 89, Catania 95123, Italy; ∥ CNR-Institute of Biomolecular Chemistry, Via Paolo Gaifami 18, Catania 95126, Italy; ⊥ Dipartimento di Scienze Chimiche, 9298 Università di Catania, Viale Andrea Doria 6, Catania 95125, Italy; # Institut des Sciences Chimiques de Rennes, CNRS UMR 6226, 27079 Université de Rennes 1, Avenue du général Leclerc, Rennes, Cedex 35042, France

## Abstract

Erbium-doped yttrium oxide nanoparticles (Er:Y_2_O_3_ NPs) with a log-normal size distribution peaking at 43 nm were synthesized and coloaded with PEG and folic acid (FA) to achieve tumor cell targeting while maintaining good water dispersibility. Structural and optical analyses (TEM, FTIR, PL, upconversion) confirmed successful functionalization without significant alterations in the fluorescence signal. In colorectal cancer cells (HCT-116), MTT assays showed >80% viability for concentrations of NPs between 0.1 and 1 μg/mL, indicating low cytotoxicity. Confocal microscopy revealed fluorescence signals consistent with potential nanoparticle internalization, although contrast was limited by cellular autofluorescence. ICP-OES quantification supported greater internalization of PEG-FA nanoparticles compared to PEG nanoparticles, confirming the role of FA in enhancing internalization. Moreover, NIR excitation (980 nm) suppressed cellular autofluorescence, suggesting the potential of these nanoparticles for high-contrast bioimaging applications.

## Graphical abstract

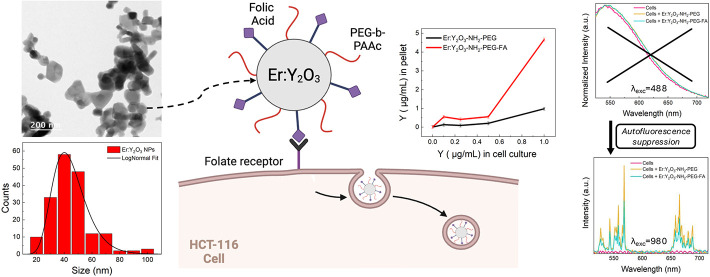

## 1 Introduction

Achieving high specificity in targeting tumor cells remains a major challenge in cancer therapy, due to tumor heterogeneity,^1^ the development of drug resistance,^2^ and the complexity of the tumor microenvironment.^3,4^ Additionally, the dynamic nature of cancer cells and the intrinsic difficulties related to drug delivery further complicate effective treatment strategies.^5^ Therefore, accurately distinguishing healthy tissue from diseased tissue is of fundamental importance so as to allow a targeted attack only on the tumor tissue, thus also minimizing systemic toxicity and improving therapeutic efficacy.

Bioimaging techniques are essential for both diagnosis and therapy of cancer. They enable detailed tumor visualization, early and accurate detection, and real-time monitoring of tumor progression and response to treatment.^6^ In this scenario, nanometric systems have been widely used. In fact, nanoparticles have outstanding optical properties and, due to their small dimensions, could easily permeate and be retained by tumor tissues and, at the same time, they are rapidly cleared by the renal excretion system.^7^ A wide variety of nanostructured probes have been explored for high-contrast bioimaging, including organic fluorophores, fluorescent dyes, quantum dots, carbon dots, metallic nanoparticles, and nanoclusters. While these systems have enabled significant advances, many of them suffer from common limitations such as photobleaching, limited penetration depth, and strong background autofluorescence under visible excitation.

Fluorescent nanoparticles^8^ and nanoclusters^9^ have been widely applied in fluorescence imaging, with confocal microscopy^10^ being one of the most commonly used techniques in this area. Moreover, the surface of nanoparticles can be readily functionalized to achieve selective interactions with cancer cells. Indeed, several tumor cell lines overexpress folate receptors,^11–13^ and for this reason, nanoparticles conjugated with folic acid (FA) can selectively bind to nonhealthy cells,^14^ enabling not only high-resolution imaging^15^ but also improving surgical resection of tumor masses.^14^ However, in conventional confocal fluorescence imaging, excitation in the visible range often results in strong cellular autofluorescence, which can severely limit signal-to-background ratios and compromise quantitative analysis, particularly at low probe concentrations. To address these limitations, rare-earth-doped yttrium oxide nanoparticles (RENPs) offer advantageous optical properties, notably upconversion, which can be exploited for deeper tissue imaging and reduced photodamage. In fact, these materials can absorb near-infrared (NIR) light and emit at shorter wavelengths in the NIR, visible (blue, green, red), or UV. The NIR excitation exploits the optical transparency window (in the NIR range of 700–1100 nm) of biological tissues, obtaining deeper light penetration and reducing photodamage effects.^16,17^ Furthermore, image contrast is often limited by background autofluorescence^18^ which can mask the emission from fluorescent probes or markers in bioimaging microscopy. Upconversion NPs^19^ can overcome this issue since NIR excitation does not induce cellular autofluorescence, thereby enabling high-contrast imaging. Despite these advantages, the effective use of upconversion nanoparticles in biological environments requires careful control of surface chemistry, colloidal stability, and targeting capability, in order to translate their intrinsic optical properties into practical imaging performance at the cellular level. A limitation of RENPs is their ceramic surface chemistry, which hinders direct use in biological environments. PEGylation^20^ is widely used to improve water dispersibility and biocompatibility; several studies^21,22^ describe its effectiveness in reducing aggregation and prolonging circulation. In fact, PEGylation reduces aggregation and improves the systemic circulation of nanoparticles (NPs), as well as the transport of actives to targeted cells, where PEGylated NPs must be internalized.^23,24^ In parallel, the introduction of targeting ligands such as folic acid enables receptor-mediated uptake, providing an additional level of selectivity that is particularly relevant for cancer cell imaging. To develop a biocompatible system that can selectively interact with tumor cells, Er:Y_2_O_3_ up-conversion nanoparticles were cofunctionalized with PEG-*b*-PAAc and Folic Acid, resulting in a system that is stable in water while also having an increased affinity for cancer cells, making it a viable candidate for bioimaging and targeted drug delivery.

This study investigates the cellular uptake of coloaded Er:Y_2_O_3_ nanoparticles (Er:Y_2_O_3_-NH_2_-PEG-FA) and PEG-only coated counterparts (Er:Y_2_O_3_-NH_2_-PEG) in human colorectal cancer cells (HCT-116). The nontoxic concentrations that still yield detectable fluorescence were determined and the intracellular localization was confirmed by confocal microscopy and ICP-OES quantification. In this respect, ICP-OES provided a very accurate quantification of the internalized nanoparticles, allowing us to single out the contribution due solely to the nanoparticles. Furthermore, the up-conversion properties of these systems have been exploited to see them also in the presence of the autofluorescence of the cells, which can be a limiting problem in bioimaging.^25^ By combining folic-acid-mediated targeting with upconversion-based imaging, this work aims to demonstrate a practical strategy for improving imaging contrast and uptake quantification in a biologically relevant cellular model.

## 2 Experimental Part

### 2.1 Materials

Y­(NO_3_)_3_·6H_2_O, Er­(NO_3_)_3_·5H_2_O were obtained as commercial reagents by Alfa Aesar (USA). Ethylenediamine (EDA), N-Chlorosuccinimide (NCS), 1-Ethyl-3-(3-(dimethylamino)­propyl)­carbodiimide (EDC), pyridine, ammonia, folic acid (FA) and DMSO were purchased from Merck (Italy). PEG-*b*-PAAc (*M*n = 5000/3200) was purchased from Polymer Source, Inc. RPMI 1640 medium, fetal bovine serum (FBS), and streptomycin/penicillin (10,000 U/mL) were obtained by Gibco, Thermo Fisher Scientific, Waltham, MA., l-glutamine from Lonza (Basel, Switzerland), and dimethyl sulfoxide (DMSO) from PanReac AppliChem. Phosphate buffered saline (PBS) was purchased from Merck (Milan, Italy) and 0.05% trypsin and 0.53 mM EDTA (1X) from Corning, Mediatech, Inc., Manassas (USA). For ICP measurements, DigiPREP SCP SCIENCE was obtained from Clark Graham Baie DUrfé, Quebec (Canada). The nitric acid for trace analysis was obtained from HNO_3_–Carlo Erba (Italy), and the Y and Er standard solution was obtained from CPAChem (Bulgaria). All solutions were prepared using Milli Q water (Resistivity > 18 MΩ·cm a 25 °C). HCT-116 cell lines, derived from primary colon tumors, were obtained from the Interlab Cell Line Collection (ICLC), an “International Repository Authority” within the IRCCS Azienda Ospedaliera Universitaria San Martino-IST Istituto Nazionale per la Ricerca sul Cancro (Genova, Italia).

### 2.2 Synthesis of Erbium Doped Yttrium Oxide Nanoparticles (Er:Y_2_O_3_)

The nanoparticles were synthesized by modifying the procedure previously for the synthesis of ultrafine yttria nanoparticles reported by S. Kassem et al.^26^ In this case 10.7990 g of Y­(NO_3_)_3_.6H_2_O and 0.9379 g of Er­(NO_3_)_3_. 5H_2_O were dissolved in 3,52 mL of Milli-Q water and NH_3_ was added until pH = 10.5 was reached. The solution was kept under stirring for 1 h at room temperature. Then, the precipitate was separated by centrifugation and washed three times (5000 rpm for 15 min each time) with Milli-Q water and dried at 100 °C for 12 h. The dried material was calcined in an air atmosphere at 1100 °C for 60 min to improve the crystallinity of the final product and finally crushed in an Agate mortar to make them homogeneous.

### 2.3 Synthesis of Ethylene Diamine Functionalized Er:Y_2_O_3_ Nanoparticles (Er:Y_2_O_3_-NH_2_)

These nanoparticles were synthesized following the procedure reported by R. Batool et al.^27^ In this case 40 mg of nanoparticles were dispersed in 5 mL of Milli-Q water and kept under sonication for an hour. Then 10 μL of EDA were added and after 24 h the obtained product was purified by centrifugation, washing two times with Milli-Q water and one with H_2_O/EtOH (50:50) and left to dry at room temperature.

### 2.4 Synthesis of Folic Acid-Functionalized Er:Y_2_O_3_ Nanoparticles (Er:Y_2_O_3_-NH_2_-FA)

Folic acid-functionalized Er:Y_2_O_3_ NPs were obtained by adding Er:Y_2_O_3_-NH_2_ to folic acid, accordingly to 27. Five mg of FA were dispersed in 5 mL of DMSO and sonicated. Subsequently, 2.32 mg of NCS and 2.4 mg of EDC were added to the folic acid solution. The system was left to react in the dark for 3 h, under stirring. Finally, 10 mg of Er:Y_2_O_3_-NH_2_ were added, and the pH was brought to 8 with pyridine. The mixture was left to react overnight. The precipitate was then washed 2 times with DMSO and 6 times with H_2_O (3500 rpm for 15 min each).

### 2.5 Synthesis of PEG-*b*-PAAc *b*- Functionalized Er:Y_2_O_3_ Nanoparticles (Er:Y_2_O_3_-NH_2_-PEG)

4.5 mg of PEG-*b*-PAAc and 6.6 mg of EDC were left to react in 5 mL of DMSO for 30 min. Subsequently, 2.3 mg of Er:Y_2_O_3_-NH_2_ and 6.6 mg of NCS were added at the same time. The pH was raised to 8 with pyridine and left to react overnight. A precipitate forms, then two centrifugation washes are carried out with DMSO (3500 rpm × 15 m). The supernatant is discarded and 5 mL of Milli-Q H_2_O were added. In water most of the nanoparticles remained in solution, even after a second spin of centrifugation.

### 2.6 Synthesis of PEG-*b*-PAAc Folic Acid-Functionalized Er:Y_2_O_3_ Nanoparticles (Er:Y_2_O_3_-NH_2_-PEG-FA)

0.0032 g of PEG were put in 5 mL of DMSO. Since each mole of PEG corresponds to 44 mol of groups COOH, the amount of reactive groups was 0.0032 g/8200 g/mol = 3.90 × 10^–7^ mol × 44 = 1.72 × 10^–5^ mol. Wanting to achieve an equimolar ratio between PEG and folic acid (n FA = n PEG), 7.6 mg of FA (1.72 × 10^–5^ mol × 441.40 g/mol) were added, together with 6.8 mg of EDC. After 30 min 1.9 mg of Er:Y_2_O_3_-NH_2_ and 6.3 mg of NCS were added at the same time, creating an equimolar ratio also for coupling agents. The pH was adjusted to 8 with pyridine and the suspension was left to react overnight. The precipitate was washed two times with DMSO by centrifugation (3500 rpm for 15 min). The supernatant was discarded and 5 mL of Milli-Q H_2_O was added, obtaining a clear solution. Following further centrifugation, no precipitate formation was observed.

### 2.7 TEM Analyses

Transmission electron microscopy analyses were carried out with a JEOL 1400 transmission electron microscope (Japan). The samples were prepared by placing a 300 mesh carbon coated nickel grids on top of a 40 μL sample droplet for 1 min (suspensions of Er:Y_2_O_3_ or Er:Y_2_O_3_-NH_2_ NPs), taking care to remove the excess liquid with paper. A 200 kV acceleration voltage was used. Particles distributions were determined from TEM micrographs using Fiji *software.*

### 2.8 FTIR–ATR Measurements

The FTIR–ATR (attenuated total reflectance) spectra were carried out using an FTIR–ATR Bruker instrument in the range 450–4000 cm^–1^, with a resolution of 1 cm^–1^ and by using a diamond crystal. All the samples were obtained by lyophilizing the solutions of the nanoparticles and of the folic acid.

### 2.9 Photoluminescence

Photoluminescence measurements were performed on a Horiba Nanolog spectrofluorometer. Measurements were performed at room temperature by depositing three 100 μL drops of the samples containing the nanoparticles on a 1 × 1 cm^2^ silicon block and allowing them to dry on a hot plate. Measurements were performed by exciting the nanoparticles at 378 nm. For cellular samples, the same procedure was followed, except that an appropriate amount of cells (in grams) was deposited to match the nanoparticle-to-cell ratios previously determined by ICP analysis after incubation experiments. In this case, excitation was performed at 488 nm, consistent with the wavelength used in the confocal microscopy measurements.

### 2.10 Upconversion

Upconversion measurements were carried out at room temperature on a home assembled spectrofluorometer, equipped with a laser operating using an excitation LED wavelength of 980 nm (incident beam at about 45° focused to approximately at 0.1 mm and max power output of 2 W), a Jobin-Yvon Triax 320 monochromator (grating 1200 grooves/mm) and a Hamamatsu photomultiplier. The analyses were carried out on the same samples prepared according to the protocol described in the photoluminescence measurements section.

### 2.11 Cell Culture

Human colorectal cancer cells (HCT-116) were cultured in RPMI 1640 medium supplemented with 10% FBS, 1% streptomycin/penicillin (10,000 U/mL), and 2 mM l-glutamine. Cells were cultivated at 37 °C and 5% CO_2_.

### 2.12 Cell Viability Assay

HCT-116 cells were seeded at 37 °C and 5% CO_2_ in a 96-well plate at a density of 1.5 × 10^4^ cells per well and starved after 24 h of seeding. Then, cells were exposed to different concentrations of PEGylated Er:Y_2_O_3_-NH_2_ NPs and Er:Y_2_O_3_-NH_2_-PEG-FA (0.1, 0.25, 0.5, and 1 μg/mL) at different time points (24–48 h) at 37 °C with 5% CO_2_. Other experimental details have been previously reported.^23^

### 2.13 ICP-OES Analyses

ICP-OES measurements were carried out to evaluate the concentration of Er:Y_2_O_3_-NH_2_-PEG and Er:Y_2_O_3_-NH_2_-FA-PEG nanoparticles (NPs) in solution. Subsequently, yttrium (Y) was quantified in treated cells to verify the nanoparticle internalization process. HCT-116 cells were seeded in 96-well plates at a density of 1.5 × 10^4^ cells per well and cultured at 37 °C in a humidified atmosphere with 5% CO_2_. After 24 h, cells were starved and subsequently incubated for 24 h with Er:Y_2_O_3_ nanoparticles at different concentrations (0.1, 0.25, 0.5, and 1 μg/mL). After incubation, cells exposed to nanoparticles and untreated cells were washed three times with 100 μL of ultrapure water to remove noninternalized nanoparticles. Cells were then detached using 50 μL of 0.05% trypsin–0.53 mM EDTA (1X) and resuspended in 100 μL of complete medium. For each tested concentration, cellular suspensions obtained from 8 independent wells were pooled together to improve statistical robustness and reduce experimental variability. The pooled samples were centrifuged (Beckman centrifuge, J-6M/E, JS 5.2 rotor), the supernatant was discarded, and the resulting cellular pellets were washed once with 100 μL of ultrapure water by centrifugation. All centrifugation steps were performed at 1100 rpm for 15 min at 20 °C. Nanoparticle suspensions and cellular pellets were mineralized using a DigiPREP SCP SCIENCE digestion system. In detail, 1 mL of nanoparticle suspension or approximately 0.020 g of cellular pellet was digested by adding 2 mL of 65% nitric acid for trace analysis and 1 mL of ultrapure water in certified low-metal HDPE vessels. Digestion was carried out at 120 °C for 1 h. Analytical blanks were processed following the same procedure. After cooling to room temperature, digested samples were diluted to a final volume of 10 mL with ultrapure water. Elemental quantification of yttrium (Y) and erbium (Er) was performed using an ICP-MS Elan DRC-e instrument (PerkinElmer, USA). Calibration was carried out using a blank and six standard solutions prepared in the same acid matrix, covering a concentration range of 1–100 μg/L for both elements. All standards and samples were spiked with rhodium as an internal standard (final concentration 25 μg/L) to correct for matrix effects and instrumental drift. Instrumental stability was monitored by ensuring that the rhodium signal intensity in the samples remained within 70–130% of that measured in the calibration blank. Quality control standards were analyzed every 10 samples and at the end of each analytical sequence, with measured concentrations within ± 10% of the nominal value (10 μg/L) for both Y and Er. Results were expressed as μg/g for cellular pellet samples and as mg/L for nanoparticle suspensions.

### 2.14 Confocal Microscopy

For confocal microscopy experiments, HCT-116 cells were seeded on chambered glass-bottom slides (8-well format) at a density comparable to that used in the viability assays and cultured at 37 °C in a humidified atmosphere with 5% CO_2_. After 24 h from seeding, cells were incubated with Er:Y_2_O_3_ nanoparticles at the same concentrations used for cytotoxicity and internalization studies. Following 24 h of nanoparticle exposure, cells were gently washed with phosphate-buffered saline to remove excess nanoparticles and fresh complete culture medium was added prior to imaging. Confocal fluorescence images were acquired using a Leica TCS SP8 confocal microscope equipped with an HCX PL APO CS2 63× oil immersion objective (NA 1.40; Leica Microsystems, Mannheim, Germany). Excitation was provided by a 488 nm laser line operated at low power to minimize photobleaching and photodamage, and fluorescence emission was collected in the 545–580 nm spectral range using a hybrid photodetector. Transmitted light images were acquired simultaneously to visualize cell morphology and confirm cell integrity during imaging. For fluorescence quantification, confocal images were analyzed by selecting regions of interest (ROIs) of identical size within intracellular areas exhibiting detectable fluorescence for each nanoparticle concentration. Multiple ROIs were analyzed for each experimental condition, and the mean fluorescence intensity was calculated. The average fluorescence intensity measured in untreated control cells was subtracted from the values obtained for nanoparticle-treated cells in order to correct for cellular autofluorescence. This comparative analysis was used to evaluate relative changes in intracellular fluorescence intensity as a function of nanoparticle concentration.

## 3 Results and Discussion

### 3.1 Synthesis and Characterization of Er:Y_2_O_3_ Nanoparticles

Since particle size strongly influences cellular internalization, a different synthetic approach than the one used in previous works^23,28^ was used, in order to obtain smaller-sized nanoparticles. The synthesis of bare Er:Y_2_O_3_ nanoparticles was adapted from the procedure reported by Kassem et al. for obtaining ultrafine Y_2_O_3_ nanoparticles.^26^ This synthesis protocol was modified by adding the correct Er/Y ratio to obtain luminescent particles.

As reported by the authors, the use of a highly supersaturated solution enabled the formation of very small particles. In our case as well, TEM microscopies (Figure 1), confirmed the presence of spherical and small-sized nanoparticles with a log-normal size distribution centered at 43 ± 1 nm.

**Figure 1:**
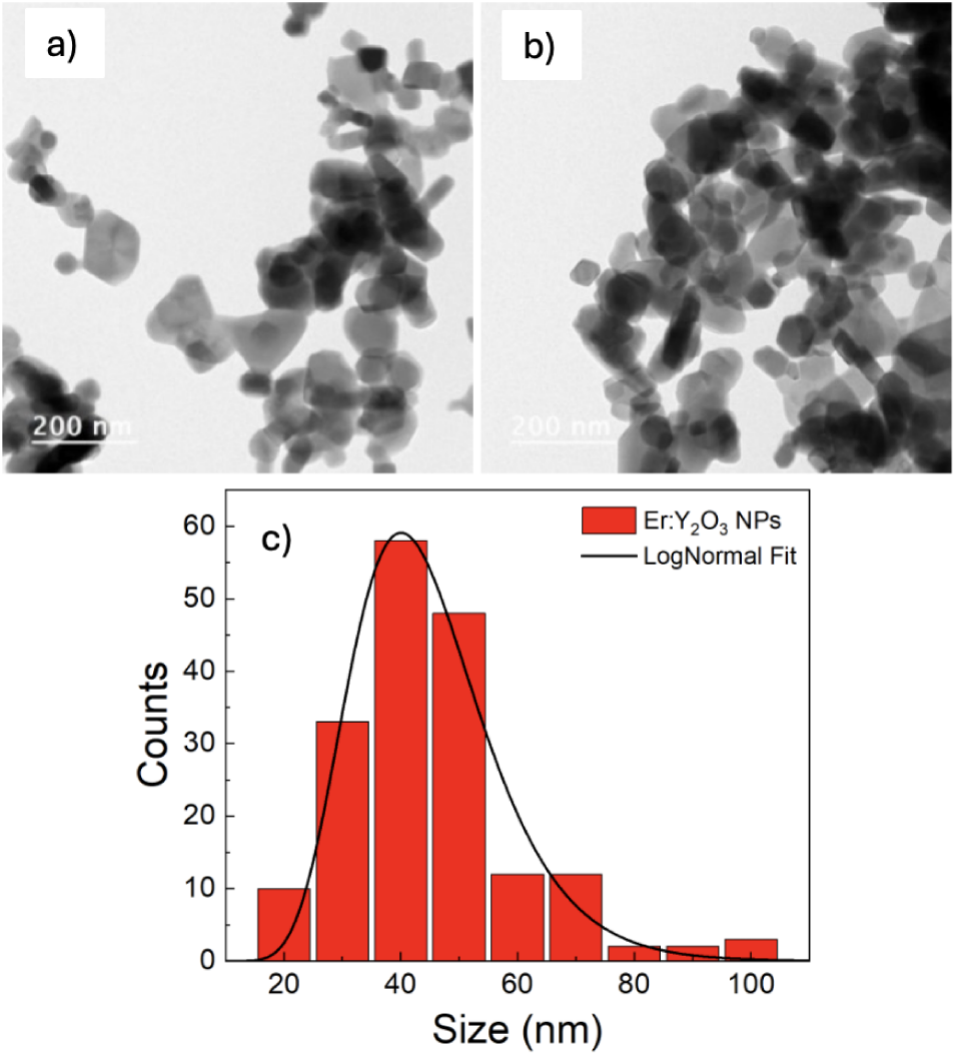
(a, b) TEM images of Er:Y_2_O_3_ NPs; (c) Particles size distribution histogram and LogNormal fit obtained by TEM images.

To evaluate the percentage of Er present in the NPs, ICP-OES measurements were carried out. The percentage of Er measured in the nanoparticles (8%) was essentially the same as previously reported,^28^ which corresponds to the optimal composition for achieving a high fluorescence signal.^29,30^ Thus, the synthesis was successfully carried out, demonstrating that the procedure reported for the obtainment of ultrafine yttria nanoparticles is also applicable for the synthesis of erbium-doped nanoparticles.

The XRD and FTIR spectra of Er:Y_2_O_3_nanoparticles synthesized by this procedure are reported in Figure 2. The XRD spectrum of Er:Y_2_O_3_ nanoparticles (Figure 2a) shows the characteristic diffraction peaks of Y_2_O_3_ and is in good agreement with the conventional cubic phase of Y_2_O_3_ with space group *Ia*3̅ (PDF Card No.: 00-079-1256). The calculated lattice constant is 10.58 Å, in agreement with that reported in the card (10.60 Å). These data confirm the same crystalline structure as the nanoparticles obtained with the other synthetic strategies.^23,28^ Furthermore, in the FTIR–ATR spectrum of the same bare nanoparticles (Figure 2b), an absorption band is observed around 500 cm^–1^ and 600 cm^–1^, corresponding to the characteristic stretching frequencies of the Y–O (metal–oxygen) bond as reported in the literature.^31,32^ The peaks centered around 1550 and 1400 cm^–1^ could be attributed to the stretching mode of the C–O bond, likely due to traces of carbonates forming on the surface of the basic oxide. The broad absorption band around 3500 cm^–1^ could be attributed to the O–H stretching vibration mode of the adsorbed moisture.

**Figure 2:**
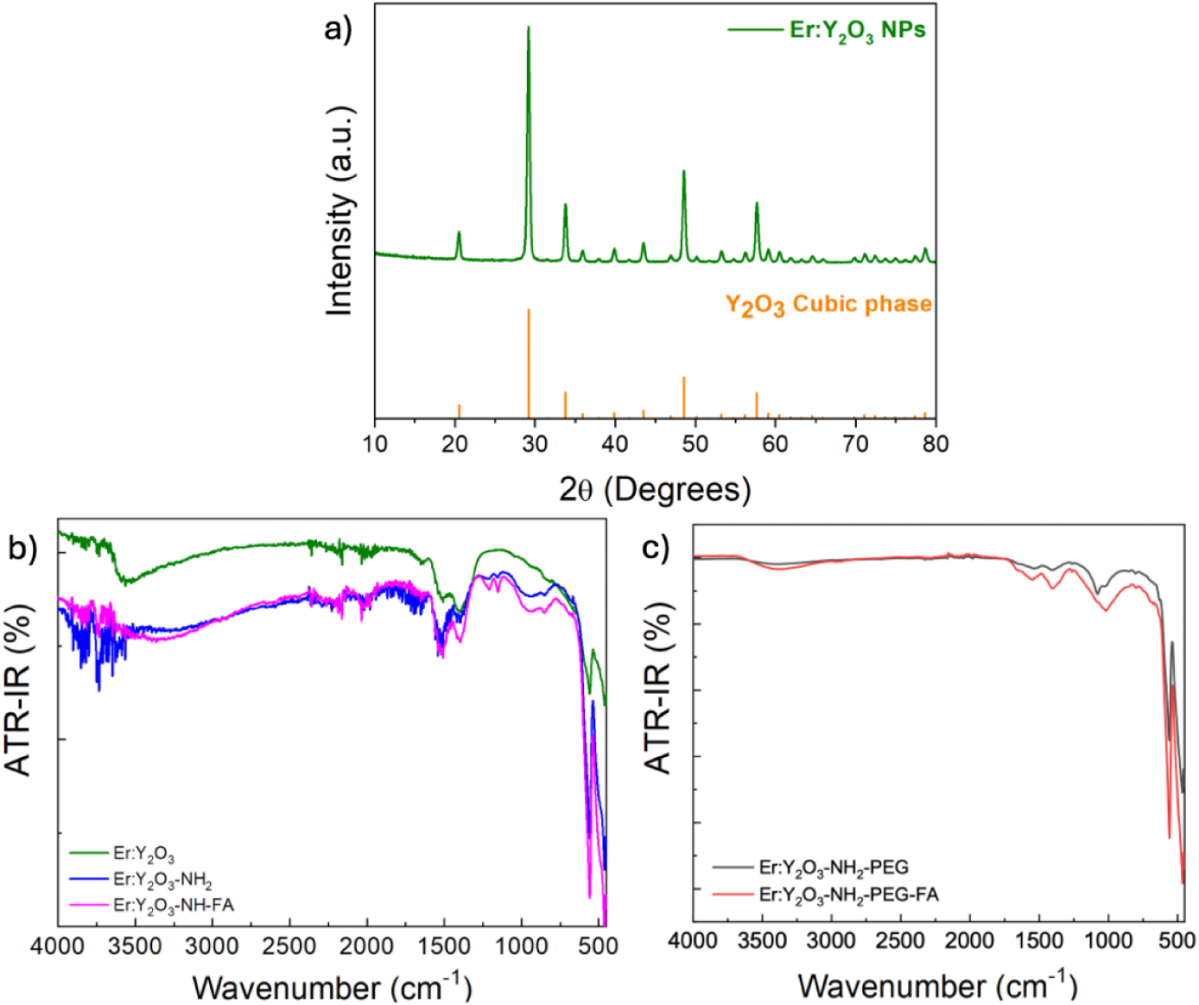
(a) XRD spectra of Er:Y_2_O_3_ NPs (green) and the characteristic diffraction peaks of the Y_2_O_3_ cubic phase (orange). FT-IR spectra of b) Er:Y_2_O_3_, Er:Y_2_O_3_-NH_2_ and Er:Y_2_O_3_-NH_2_-FA NPs and c) Er:Y_2_O_3_-NH_2_-PEG and Er:Y_2_O_3_-NH_2_-PEG-FA NPs.

The Er:Y_2_O_3_-NH_2_ were synthesized by following the procedure reported in ref^27^. The functionalization was successful as demonstrated by the FTIR–ATR spectra reported in Figure 2b. In fact, by comparing the spectrum of Er:Y_2_O_3_-NH_2_ nanoparticles with that of the bare ones, a broad and more intense absorption band is observed between 4000 and 3000 cm^–1^ attributed to N–H stretching, together with a band in the range of 1500–1700 cm^–1^, assigned to the primary amine N–H bending.

To provide the nanoparticles with targeting ability toward cancer cells overexpressing folate receptors, they were functionalized with folic acid. Folic acid-functionalized Er:Y_2_O_3_ NPs were synthesized as reported in ref^27^ and their FTIR–ATR spectrum, reported in Figure 2b, confirmed that the functionalization was successful. In fact, in the 1750 to 1000 cm^–1^ region the absorption bands are more intense than in the bare and aminated nanoparticles and, since these bands can be attributed to the amine, amide and carboxylic groups of folic acid, this last was successfully bonded on the nanoparticles surface. However, these NPs are not soluble in water and therefore are not useful for the purpose of this work. To overcome this difficulty, a PEGylation procedure and a coloading procedure of PEG and folic acid on the particles themselves were developed and optimized. In fact, PEGylation is a procedure widely used to make inert materials soluble. To this aim, different PEG/NPs ratios were tested, and the block copolymer was covalently bound by its −COOH groups to the NH_2_ groups of the Er:Y_2_O_3_-NH_2_ NPs. This was confirmed by the FTIR spectra reported in Figure 2c. In fact, the spectrum of Er:Y_2_O_3_-NH_2_-PEG shows a less intense signal in the 1750–1500 cm^–1^ region, consistent with the disappearance of the N–H bending modes, whereas a new absorption in the 1300–1200 cm^–1^ region appears, corresponding to the coupled C–N stretching and N–H bending motions, characteristic of the amide III vibration as recently reported in a DFT study.^33^ Subsequently, singled out the best PEG/NPs ratio, this was used in a coloading procedure in which FA and PEG-*b*-PAAc were simultaneously added to the solution containing the aminated NPs and the coupling agents. In this case the product precipitated in DMSO but was soluble in water, as described in the experimental section. Thus, this strategy allowed us to obtain nanoparticles functionalized with folic acid and water-soluble. The comparison of the FTIR spectrum of the Er:Y_2_O_3_-NH_2_-PEG-FA with that of Er:Y_2_O_3_-NH_2_-PEG shows that either FA and PEG were bonded on the nanoparticles surface. In fact, also in Er:Y_2_O_3_-NH_2_-PEG-FA spectrum, an absorption band is observed around 500 cm^–1^ and 600 cm^–1^, confirming the Y–O (metal–oxygen) bond stretching. The peaks centered around 1550 and 1400 cm^–1^, which are particularly intense, can be attributed to Folic Acid whose two carboxylic groups, upon conjugation with the NPs, lead to the formation of an amide group, with stretching vibrations also detected in this region.There is also a well-defined absorption band between 3500 cm^–1^ and 3000 cm^–1^ attributed to the O–H of folic acid, that is not visible in the PEGylated one. The PL spectra of all the synthesized nanoparticles are reported in Figure 3a–d.

**Figure 3:**
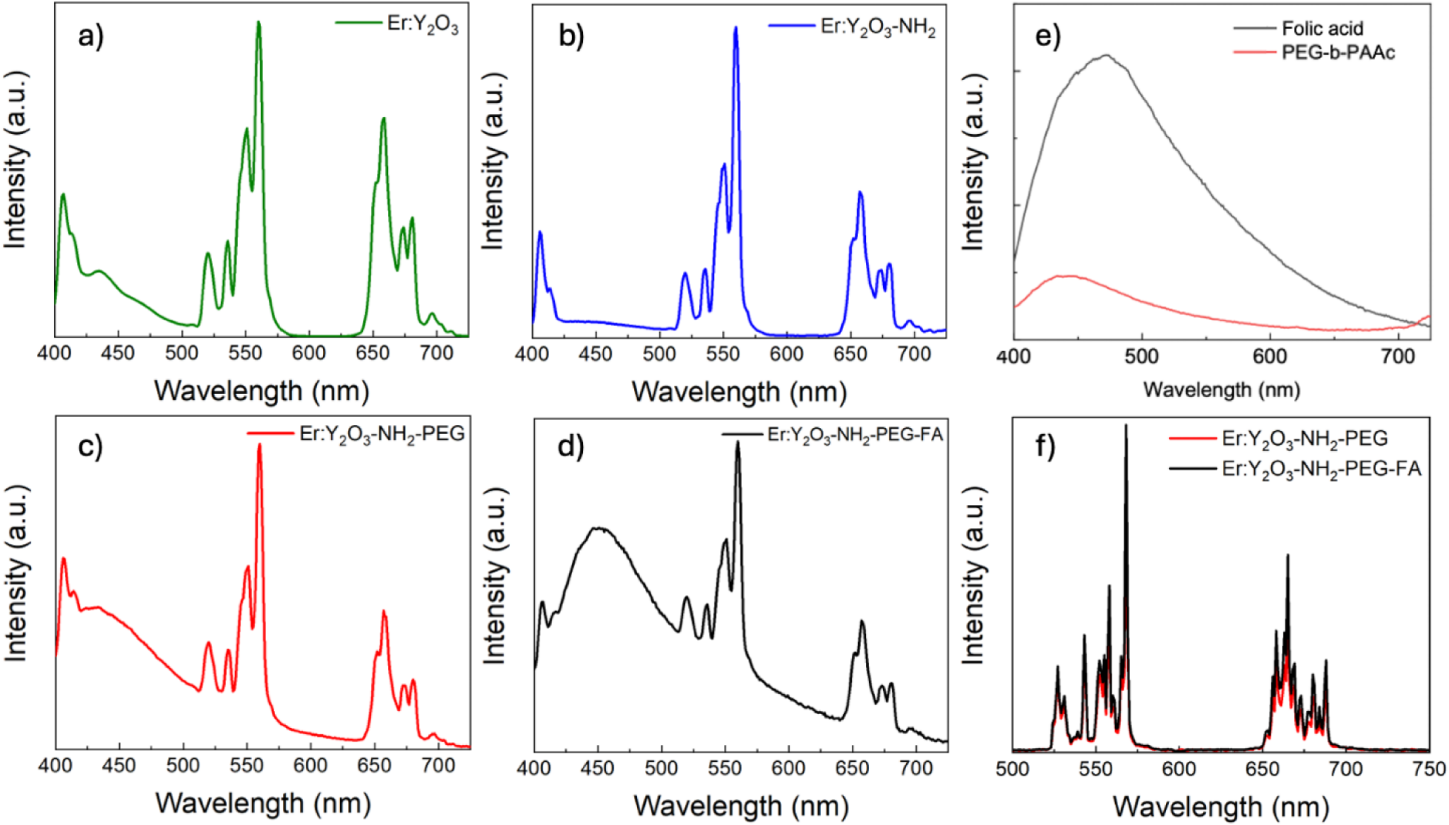
PL downconversion spectra of a) Er:Y_2_O_3_, b) Er:Y_2_O_3_-NH_2_, c) Er:Y_2_O_3_-NH_2_-PEG, d) Er:Y_2_O_3_-NH_2_-PEG-FA NPs and e) Folic Acid and PEG-*b*-PAAc; λ_exc_ = 378 nm. f) PL upconversion spectra of Er:Y_2_O_3_-NH_2_-PEG (red) and Er:Y_2_O_3_-NH_2_-PEG-FA NPs (black); λ_exc_ = 980 nm.

All spectra show typical erbium peaks, suggesting that the surface functionalization does not affect the crystalline matrix and, consequently, does not alter the luminescence properties of the systems, making them suitable for our study. In addition, a band in the 400–500 nm region is observed in the spectra of Er:Y_2_O_3_-NH_2_-PEG and Er:Y_2_O_3_-NH_2_-PEG-FA NPs, which can be attributed to the presence of PEG and FA. This band is more intense in the spectrum of the cofunctionalized Er:Y_2_O_3_-NH_2_-PEG-FA NPs, reflecting the stronger contribution of folic acid. Figure 3e shows the photoluminescence spectra of PEG and FA as controls.

The upconversion spectra of Er:Y_2_O_3_-NH_2_-PEG and Er:Y_2_O_3_-NH_2_-PEG-FA NPs are reported in Figure 3f. The spectra show that both nanoparticles exhibit the same emission lines, confirming once again that functionalization does not influence the intrinsic properties of these systems which have a characteristic emission pattern in the visible region of the spectrum when excited in the IR (980 nm).

### 3.2 Study of Toxicity and Internalization inside HCT-116 Cancer Cells

The cytotoxicity of both bare and surface-functionalized nanoparticles was evaluated in order to assess their suitability for in vitro imaging experiments. An MTT assay was carried out after incubation of HCT-116 cells with Er:Y_2_O_3_-NH_2_-PEG and Er:Y_2_O_3_-NH_2_-PEG-FA NPs at different concentrations (0.1, 0.25, 0.5, and 1 μg/mL) and at two incubation times (24 and 48 h). The results (Figure 4a) show that cell viability remained relatively high at all concentrations of Er:Y_2_O_3_-NH_2_-PEG nanoparticles. Small fluctuations in viability were observed, but no clear dose-dependent trend was evident. These variations are likely due to experimental or biological variability rather than a concentration-related cytotoxic effect. After 48 h, viability shows values slightly higher or equivalent compared to the data obtained at 24 h, suggesting that the vitality of the cells is partially restored, with a relatively high viability.

**Figure 4:**
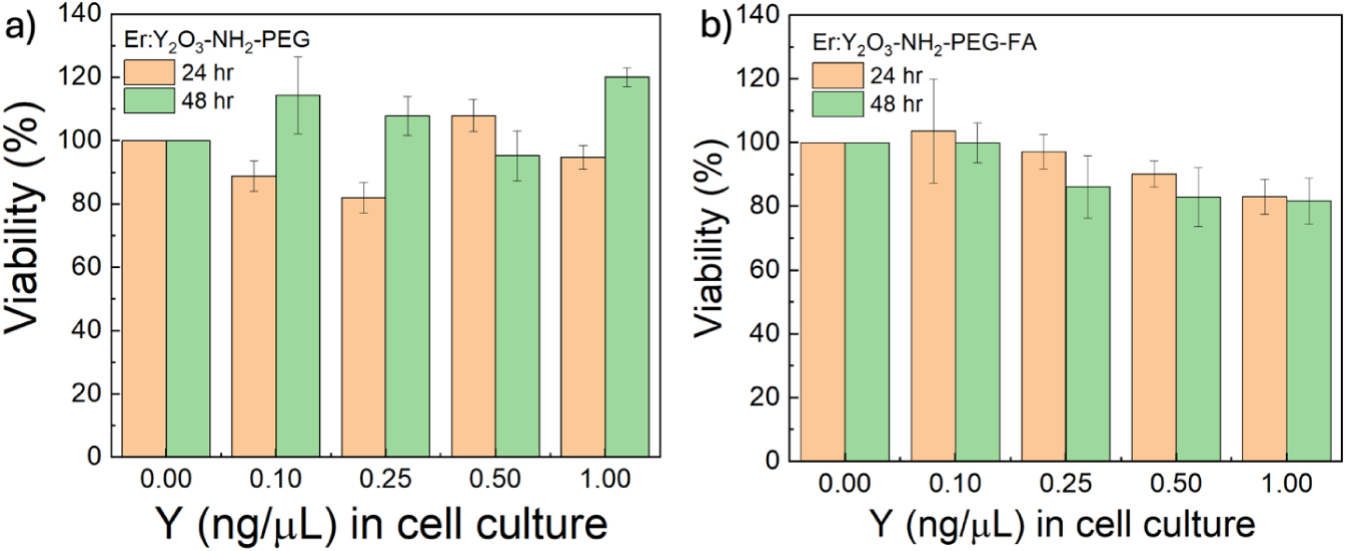
Viability percentage (%) of HCT-116 incubated with of a) Er:Y_2_O_3_-NH_2_-PEG and b) Er:Y_2_O_3_-NH_2_-PEG-FA NPs at different concentrations (0.1, 0.25, 0.5, and 1 μg/mL): yellow bars at 24 h and green bars at 48 h. Only the majority component of NPs (yttrium) was used to define the concentration.

In the case of Er:Y_2_O_3_-NH_2_-PEG-FA nanoparticles (Figure 4b), cell viability showed a more pronounced reduction with increasing concentration compared to nanoparticles containing only PEG. At 24 h, viability remained above 80% for all concentrations tested, but a progressive decrease was observed at higher doses, with values approaching 80% at 1 μg/mL. A similar trend was evident after 48 h, where the reduction in viability became more pronounced. These results suggest that the presence of folic acid may enhance nanoparticle internalization by cells, leading to a stronger cytotoxic response. However, cell viability remained above 80% at all concentrations and time points tested, making these concentrations suitable for in vitro bioimaging and internalization studies in the selected cellular model.

The NPs internalization was verified at 24 h for both NPs. Thus, the solutions of Er:Y_2_O_3_-NH_2_-PEG and Er:Y_2_O_3_-NH_2_-PEG-FA NPs particles at different concentrations (0–1 μg/mL) were incubated for 24 h with HCT-116 cells and were then observed with a confocal microscope. The confocal microscope images of control cells and cells incubated with NPs at the concentration of 1 ng/μL are reported in Figure 5.

**Figure 5:**
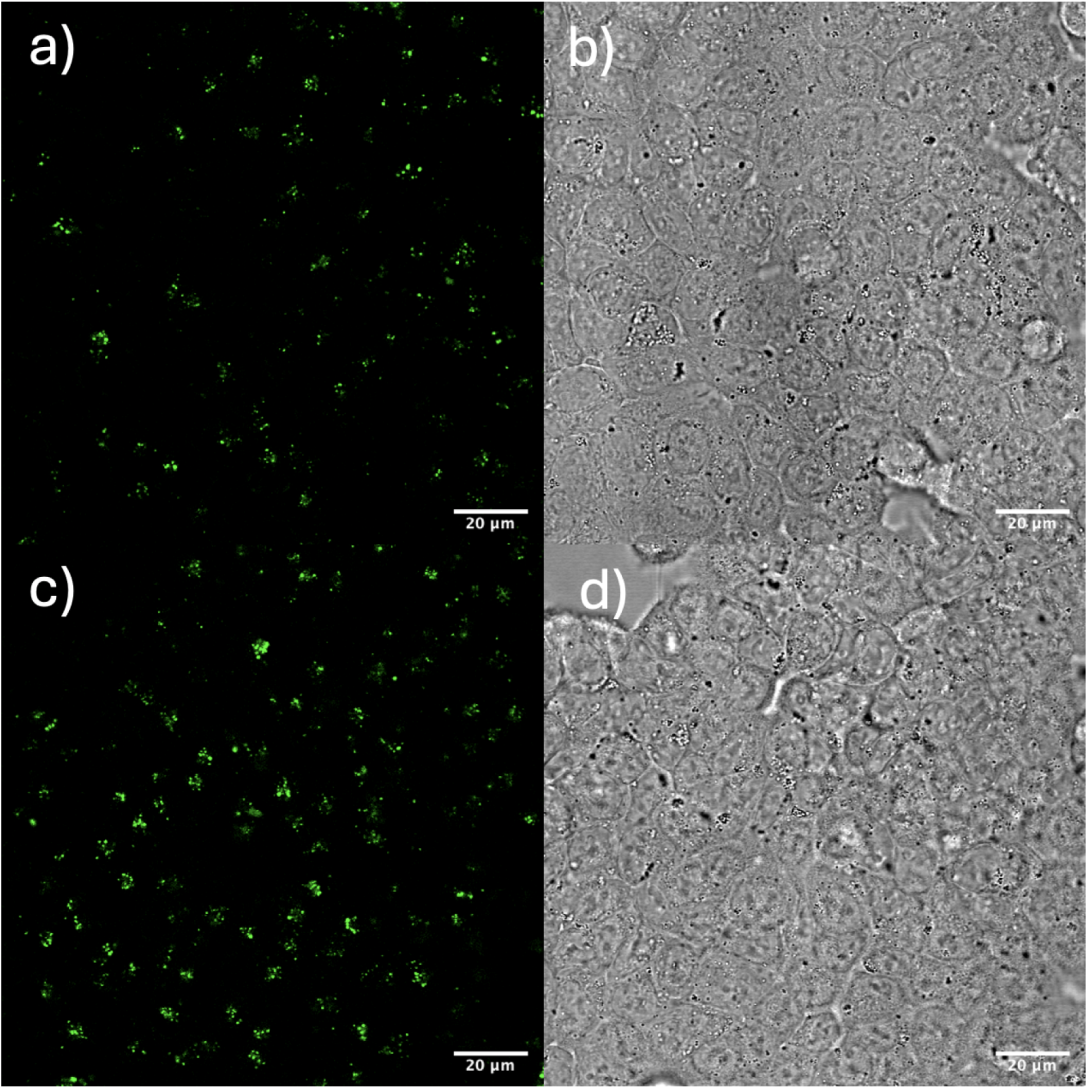
Confocal microscopy images in a,c) fluorescence and b,d) bright-field channel of HCT-116 cancer cells incubated with with Er:Y_2_0_3_-NH_2_-PEG-FA NPs at a,b) 0 μg/mL, and c,d) 1 μg/mL after 24 h of incubation. Scale bar: 20 μm; λ_exc_ = 488 nm.

The confocal images show that the fluorescence is higher in the cells incubated with the NPs, although the contrast remains reduced due to the intrinsic autofluorescence of the cells, which overlaps with the emission wavelength of the nanoparticles. The observed fluorescence does not originate from the cell walls but from within the cells, suggesting that the nanoparticles have been internalized. To overcome the limitations related to low visual contrast, the confocal images were analyzed quantitatively, as described in the Materials and Methods, in order to obtain a more objective data of the variation in fluorescence as a function of the NPs concentration. The results, reported in Figure 6a, show an increase in fluorescence intensity with increasing concentration for both Er:Y_2_O_3_-NH_2_-PEG and Er:Y_2_O_3_-NH_2_-PEG-FA NPs; however, in the case of particles cofunctionalized with PEG and FA the increase is higher. Finally, to evaluate and confirm the internalization of NPs into the cells, ICP-OES measurements were performed to measure the amount of Y present in the cellular pellets incubated with NPs at different concentrations, and the results are shown in Figure 6b. Since the matrix of the nanoparticles is Y_2_O_3_, measuring the amount of Y, gives us an indirect measure of the amount of NPs. All the NPs present outside the cells were eliminated by washing, and thus, the concentration of Y present in the pellets was equivalent to the concentration of Y inside the cells, reflecting the number of internalized NPs. The results (Figure 6b) show an increase in the concentration of Y present inside the pellets consistent with the increase. Furthermore, the higher Y concentration revealed by ICP-OES in pellets incubated with Er:Y_2_O_3_-NH_2_-PEG-FA NPs suggests greater internalization compared to the Er:Y_2_O_3_-NH_2_-PEG ones. This result was expected, since HCT-116 cancer cells overexpress folate receptors on their membrane, which can interact with folate-targeted nanoparticles and promote their uptake through receptor-mediated endocytosis.

**Figure 6:**
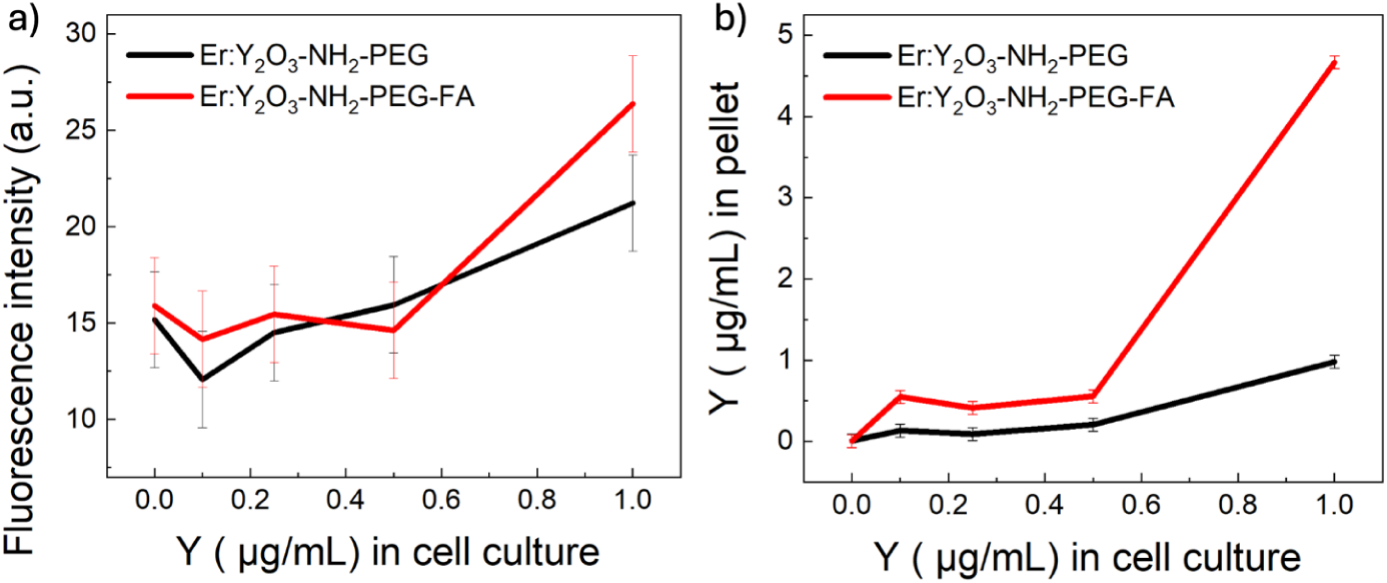
(a) Fluorescence intensity obtained from the analysis of confocal images; λ_exc_ = 488 nm. b) Concentration of yttrium measured in pellets by ICP-OES versus concentrations of yttrium in the incubation medium for cells incubated with Er:Y_2_O_3_-NH_2_-PEG and Er:Y_2_O_3_-NH_2_-PEG-FA NPs.

ICP measurements provided more reliable results, with much lower error than fluorescence quantification by confocal microscopy, which exhibits high baseline noise due to cellular autofluorescence. To overcome this limitation and obtain images with improved contrast, the upconversion properties of these nanosystems were exploited. The downconversion and upconversion spectra of the cells alone and in the presence of the Er:Y_2_O_3_-NH_2_-PEG and the Er:Y_2_O_3_-NH_2_-PEG-FA NPs are reported in Figure 7. In Figure 7a, the spectra of cells alone or in the presence of NPs show a very similar trend, with nearly overlapping normalized signals. Although an optimal wavelength was used for NPs excitation (488 nm), their characteristic emission is masked by the intense cellular autofluorescence. On the contrary, in Figure 7b, analyzing the same samples but with NIR excitation (980 nm), the cellular autofluorescence is completely eliminated and only the upconversion signal of the nanoparticles is visible. This effect allows for a significantly higher contrast by isolating the contribution of the NPs from that of the cells. These spectral characteristics, together with their ability to be internalized into cells, highlight the potential of these nanoparticles for bioimaging and targeted therapeutic applications.^34–36^

**Figure 7:**
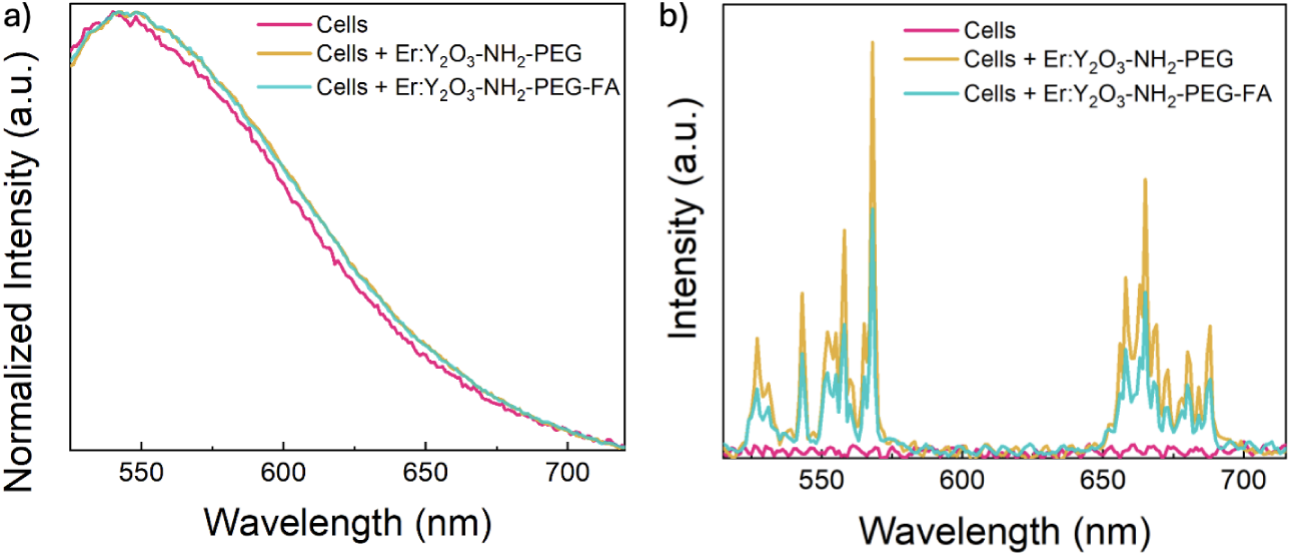
(a) Downconversion (λ_exc_ = 488 nm) and b) upconversion (λ_exc_ = 980 nm) PL spectra of cells incubated with Er:Y_2_O_3_-NH_2_-PEG and Er:Y_2_O_3_-NH_2_-PEG-FA NPs.

## 4 Conclusions

In this work, Er:Y_2_O_3_ nanoparticles were synthesized and subsequently functionalized with PEG and folic acid (FA). This functionalization was performed to enhance cellular internalization through FA recognition of folate receptors, overexpressed in the HCT-116 colorectal cancer cell line. Coloading with PEG improved the stability of the NPs in solution by enhancing their hydrophilicity. TEM analysis confirmed the formation of spherical nanoparticles with a characteristic diameter of 43 ± 1 nm.

The fluorescence properties of the bare and functionalized NPs were studied, revealing that, despite surface modifications, the intrinsic luminescence of Er^3+^ ions is maintained in the NPs. Photoluminescence in the visible range was observed in both downconversion and upconversion, with excitation at 378 and 980 nm, respectively.

Cellular experiments demonstrated low cytotoxicity of both Er:Y_2_O_3_-NH_2_-PEG and Er:Y_2_O_3_-NH_2_-PEG-FA NPs, even at the highest concentration tested (1 μg/mL, 24–48 h of incubation). Internalization studies, conducted by confocal microscopy and quantified by ICP-OES analysis of intracellular yttrium, showed a progressive increase in internalization with increasing NPs concentration incubated within the cell culture. Importantly, NPs functionalized with FA showed greater internalization than NPs functionalized with PEG alone, confirming the targeting role of folic acid.

However, confocal analysis showed strong autofluorescence in HCT-116 cells, which, when excited at 488 nm, re-emit in the same spectral range as the NPs, thus decreasing image contrast. To overcome this problem, the NPs were excited at 980 nm to exploit their upconversion properties. Since the cells do not absorb in the near-infrared region and do not have upconversion properties, this approach effectively eliminated background noise, allowing only the fluorescence signal from the NPs to be obtained.

These results pave the way for the use of infrared-excited confocal microscopy to exploit the intrinsic upconversion properties of Er:Y_2_O_3_ NPs. This approach allows the detection of very low concentrations of NPs within the cells, improving contrast and reducing phototoxicity. In addition to bioimaging, the ability to selectively functionalize and track Er:Y_2_O_3_-NH_2_-PEG-FA NPs opens promising prospects for drug delivery, super-resolution microscopy, and intracellular tracking in cancer research.
